# Primary congenital glaucoma surgery: outcomes and visual function

**DOI:** 10.1007/s10792-021-01957-0

**Published:** 2021-07-23

**Authors:** Elena Gusson, Francesca Chemello, Rosa Longo, Elia Franzolin, Roberta Vesentini, Giuseppe Verlato, Giorgio Marchini

**Affiliations:** 1 Department of Neuroscience, Biomedicine and Movement Sciences, University Of Verona, Eye Clinic, P.le L.A. Scuro 10, 37134 Verona, Italy; 2 Department of Diagnostic and Public Health, University Of Verona, Verona, Italy

**Keywords:** Childhood glaucoma, Visual acuity, Glaucoma surgery, Intraocular pressure, Axial length

## Abstract

**Purpose:**

To assess the long-term visual outcomes of children with PCG, irrespective of the type of surgical procedure, and to create visual acuity curves to help in predicting the development of visual function in these patients. The secondary aim is to identify associated factors for visual decline or loss, highlighting differences between neonatal and infantile subgroups.

**Methods:**

The medical records of pediatric glaucoma patients from 1996 to 2017 at the University Hospital of Verona (Verona, Italy) were retrospectively reviewed. Visual acuities, surgeries, PCG subtype and etiology of vision impairment were recorded. Statistical analyses were performed to detect factors associated with vision decline.

**Results:**

Sixty-seven eyes (40 patients) were included in the study. Developmental predictive curves of visual acuity showed that children with infantile PCG had a better visual outcome than children with neonatal PCG at each step of follow-up. A good-to-moderate VA (< 1 LogMAR) was achieved in 56 eyes (83.6%), while 11 eyes (16.4%) had poor VA (≥ 1 LogMAR). The age at onset, sex, number of surgeries, intraocular pressure (IOP) control (with or without antiglaucoma drugs), axial length (AL) and corneal opacities were statistically associated with vision impairment (*p* < 0.01). The main cause of visual impairment was amblyopia.

**Conclusions:**

Visual outcomes of PCG significantly correlate with the age at diagnosis. Although a good long-term IOP control can often be achieved in PCG, often the visual acuity remains below the lower limits of the normal range. Poor vision in childhood is related to global developmental problems, and referral to third-level services should not be delayed to prevent vision impairment. In this regard, visual acuity curves can be a useful tool for the consultant ophthalmologist to define the visual development of children affected by PCG.

## Introduction

Childhood glaucoma is a rare disease, associated with increased intraocular pressure (IOP), whose natural evolution is blindness [1, 2]. This condition can be classified as primary congenital glaucoma (PCG) or secondary glaucoma: PCG is due to trabeculodysgenesis, while secondary glaucomas can occur as a consequence of congenital or acquired ocular or systemic disorders [2–6]. PCG is the most common form of childhood glaucoma [7]. Typical presenting symptoms are epiphora, photophobia and blepharospasm. Buphthalmos (globe enlargement), edema and haze of the cornea with rupture of the Descemet’s membrane (Haab’s striae) and progressive excavation of the optic nerve (ON) are common features of PCG [8]. PCG is a difficult disease to treat, and the outcome can often be unsatisfactory [9]. Treatment may involve multiple operations in the first years of life [10–13]. Visual loss and restriction of visual field secondary to PCG may occur as a consequence of ON damage, corneal opacities, cataracts and amblyopia. To the best of our knowledge, to date there are not predicting curves of the development of visual acuity in children affected by PCG. The purpose of this study is to assess the long-term visual outcomes of children with PCG to create visual acuity curves to help the consultant ophthalmologist in predicting the development of visual function in these patients. The secondary aim is to identify associated factors for visual decline or loss in the study cohort and whether there are differences between neonatal and infantile groups.

## Methods

Patients diagnosed with PCG who were assessed and surgically treated between December 1996 and June 2017 at the Glaucoma Unit of Verona University Hospital were included in the study. The study protocol was previously approved by the Ethics Committee of our Institution. All data were collected anonymously and following the ethical standards of the Declaration of Helsinki. All the patients underwent a clinical examination in the Pediatric Ophthalmology Unit of our Institution. PCG was diagnosed based on clinical findings, following the criteria proposed by the *Childhood Glaucoma Research Network* (CGRN) [14]. All patients having retinal or inflammatory ocular comorbidities or systemic syndromes were excluded. Subcategory of PCG (neonatal, < 1 month of age, or infantile, > 1 month of age) according to CGRN [14], intraocular pressure (IOP), IOP control (with or without medication), horizontal corneal diameter (HCD), corneal transparency, axial length (AL), ON damage (defined as cup-to-disk ratio > 0.5), number of administered drugs, number of surgical procedures and best-corrected visual acuity (BCVA) were analyzed. The IOP was measured by hand-held applanation tonometer (Perkins tonometer). Pediatric ophthalmologic assessment, including visual acuity evaluation, cycloplegic refraction and amblyopia management, was performed following the “*Pediatric Eye Evaluation Preferred Practice Patterns*” of the American Academy of Ophthalmology [15, 16]. VA was assessed using age-appropriate tests, starting from Teller acuity cards for children under 3 years of age and Lea symbol charts for children above 3 years old but preschool. The Snellen acuity charts were used for literate children. Spherical equivalent of refraction (SER) was used to analyze the refractive error. Myopia and hyperopia were considered when SER exceeded one diopter. This study evaluates the BCVA of both eyes separately and the prescribed optical correction. To understand the factors associated with low visual development, the sample was divided into two groups. Group I (within the range for age) included eyes with a VA comprised in the 95% (± 2SD) prediction interval for age; Group II (under the range for age) included eyes with a VA inferior to prediction interval for age (< −2 SD) [17].

### Statistical analyses

Categorical variables were reported by frequency with percentage. The normality of data was assessed with the Shapiro–Wilk test. Normally distributed quantitative data were reported by mean with SD, not normally distributed quantitative data by median with interquartile range (IQR). *P*-value was computed by Fisher’s exact test for nominal variables and by Wilcoxon–Mann–Whitney rank sum test for quantitative variables. Statistical analysis was carried out with Stata software version 14 (StataCorp, College Station, TX). A *p*-value <0.05 was considered significant^.^

## Results

**Fig. 1:**
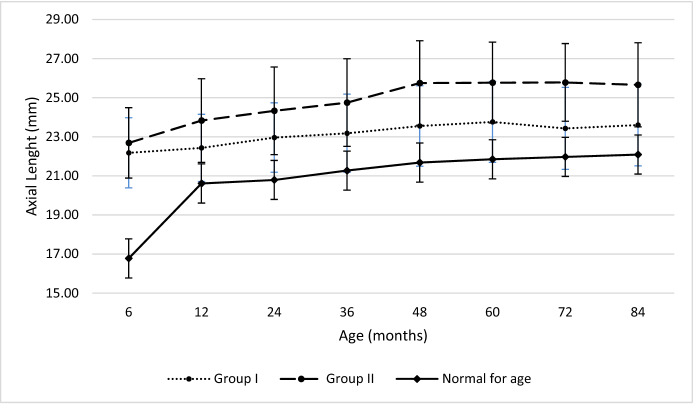
Axial length (AL) with increasing age during follow-up in childhood glaucoma as compared to age-matched normal value

**Fig. 2:**
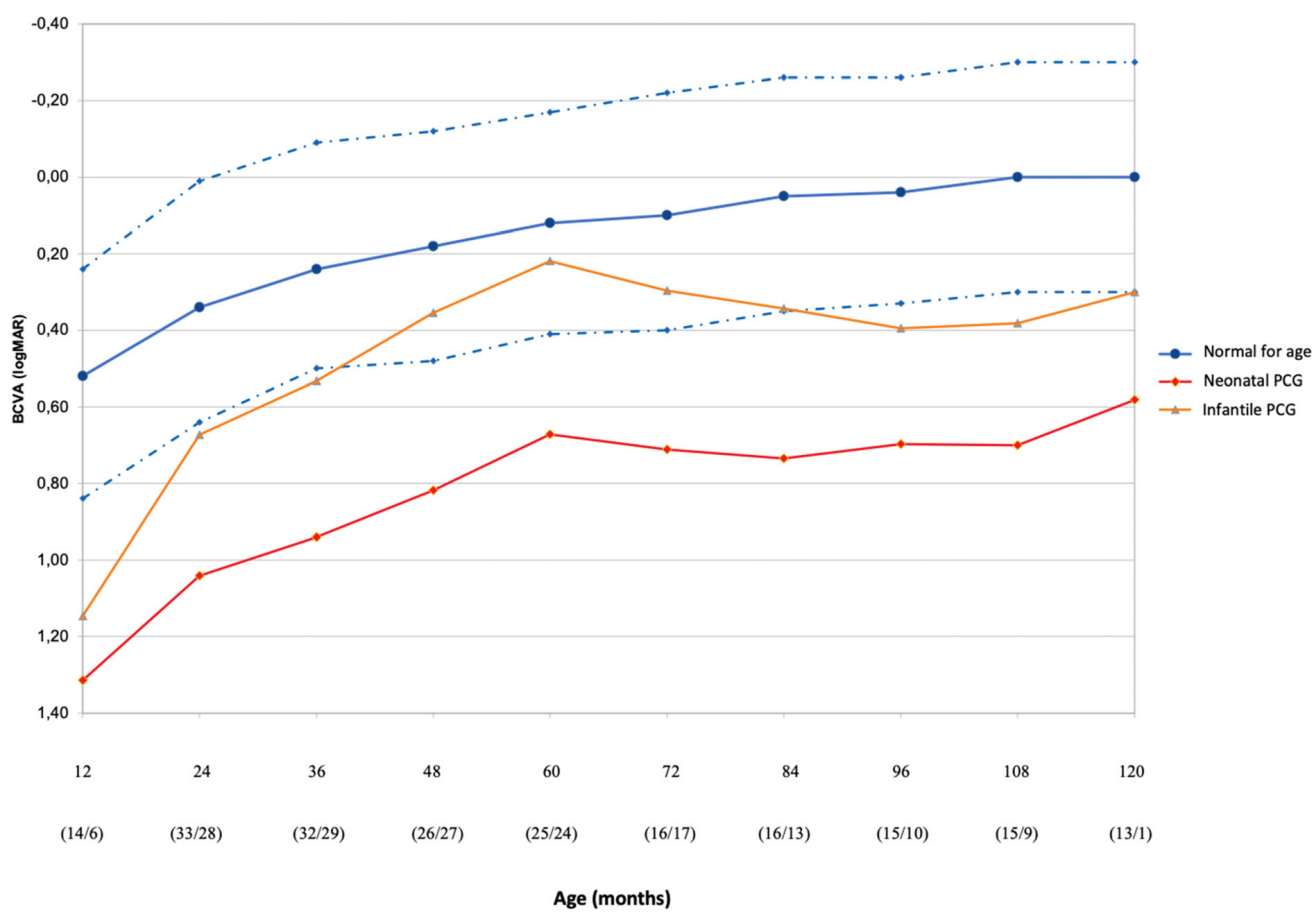
BCVA with increasing age during follow-up in childhood glaucoma as compared to age-matched normal value

Sixty-seven eyes (40 patients) were included in the study. The demographic data are presented in Table 1. Table 2 shows the clinical features of patients included in the study making a comparison between the two PCG subtypes (neonatal and infantile). The mean IOP at presentation was 23.1 ± 4.0 mmHg; at the final assessment, the mean IOP was 13.0 ± 3.0 mmHg. The percentage of reduction was 43.7% (*p* < 0.0001). No significant difference in IOP after surgery was found between different groups (*p* = 0.598). The mean AL was 20.72 ± 2.11 mm and 22.97 ± 2.12 mm at presentation and 24.66 ± 2.29 mm and 24.33 ± 2.32 mm at the final assessment, respectively, for the neonatal and infantile group. The AL at the end of follow-up was out of upper limits for age for all the glaucoma subtypes (Fig. 1) [17]. At the last consultation, the percentage of AL growth was 18.7% in the neonatal and 5.9% in the infantile. The overall rate of corneal opacity, ON damage and IOP-lowering drugs is listed in Table 3. Age at onset (*p* = 0.014), sex (*p* = 0.028), number of surgeries (*p* < 0.001), need of IOP-lowering therapy (*p* < 0.001), AL at the end of the follow-up (*p* = 0.001) and corneal opacities at baseline (*p* = 0.016) significantly differ between eyes with normal and under the range VA for age (Group I vs Group II), as shown in Table 3. Myopia was the predominant refractive error, occurring in 66.7% of eyes. The distribution of SER at the latest visit shows a significant myopic shift among the eyes with a VA under the range for age compared to the ones with a normal VA for age (*p* = 0.001). Figure 2 shows the development of VA in eyes affected by PCG comparing it to that of normal eyes of children of the same age [17]. In the neonatal group, VA was below lower normal limits at every step of follow-up. VA was within lower normal limits in the first 3 years of age and within the normal range for age after 5 years for the infantile group. A good-to-moderate VA (< 1 LogMAR) was achieved in 56 eyes (83.6%), while 11 eyes (16.4%) had poor VA (≥ 1 LogMAR). Causes of severe visual impairment (VA > 1 LogMAR) were optic neuropathy in 4 eyes, anisometropic/refractive amblyopia in 2 eyes, strabismus in 2 eyes, 2 eyes had no light perception due to total choroidal detachment secondary to surgical procedures, and one eye deprivation amblyopia due to corneal opacification.

**Table 1:** Demographic and clinical features of 40 patients (67 eyes) with primary congenital glaucoma (PCG)

	Neonatal PCG	Infantile PCG	Total
Patients, n	18	22	40
Eyes, n	33	34	67
Sex (M/F), n (%)	6:12 (33:67)	14:8 (64:36)	20:20 (50:50)
Ethnicity (Caucasian/African), n (%)	17:1 (94:6)	19:3 (86:14)	36:4 (90:10)
Bilateral: unilateral, n (%)	15:3 (83:17)	15:7 (68:32)	30:10 (75:25)
Age at first surgery (mo), mean ± SD	1.8 ± 1.9	8.8 ± 4.1	5.7 ± 4.8
Surgeries, median (range)	2.5 (2–4)	2 (1–2)	2 (1.75–3)
Follow-up duration (mo), mean ± SD; median	114.8 (59.9–163.3)	66.8 (49.9–94.4)	82.3 (54.8–114.4)

**Table 2:** Clinical characteristics at the end of follow-up in the PCG groups

	Neonatal	Infantile	Total
IOP (mmHg), mean ± SD	13.2 ± 3.0	12.8 ± 3.0	13.0 ± 3.0
AL (mm), mean ± SD	24.66 ± 2.29	24.33 ± 2.32	24.49 ± 2.29
Corneal opacity, yes/no (%)	36.4: 63.6	11.8: 92.2	23.9: 76.1
Haab striae, yes/no (%)	18.1: 81.9	47.1: 52.9	32.8: 67.2
Corneal diameter, mean ± SD	13.02 ± 0.85	13.69 ± 0.77	13.36 ± 0.87
ON damage, yes/no (%)	36.4: 63.6	68.8: 41.2	50.7: 49.3
Patching, yes/no (%)	50: 50	61.9: 38.1	56.4: 43.6
Strabismus (yes/no), n	50: 50	19.0: 81.0	35.9: 64.1
SER, mean ± SD	4.83 ± 3.67	3.85 ± 3.59	4.35 ± 3.63
Medical therapy, yes/no (%)	48.5: 51.5	26.5: 73.5	37.3: 62.7

*IOP* intraocular pressure, *AL*: axial length, *ON* optic nerve, *SER* spherical equivalent of refraction

**Table 3:** Clinical features of patients with normal BCVA for age and under-normal range for age

BCVA			
	Normal for age	Under-range for age	*p*-value
Eyes, n (%)	37(55.2)	30(44.8)	–
Follow-up duration, months	84.3 ± 52.3	88.0 ± 66.5	.800
Gender, M/F (%)	64.9/35.1	36.7/63.3	**.028**
Ethnicity, Caucasian/African (%)	91.9/8.1	86.7/13.3	.691
Laterality, bi/unilateral (%)	86.5/13.5	83.3/16.7	.743
PCG, neonatal/infantile (%)	35.1/64.9	66.7/33.3	**.014**
Number of surgeries	1 (1–1)	2 (1–2)	** < .001**
IOP at baseline (mmHg)	22.8 ± 4.3	23.4 ± 3.6	.594
IOP at final follow-up (mmHg)	12.5 ± 2.9	13.5 ± 3.0	.178
AL at baseline (mm)	21.85 ± 1.99	21.87 ± 2.83	.967
AL at final assessment (mm)	23.73 ± 2.01	25.43 ± 2.30	**.001**
Corneal opacity at baseline, Yes/No (%)	59.5/40.5	86.7/13.3	**.016**
CDR at baseline	0.44 ± 0.17	0.51 ± 0.21	0.231
Drugs at baseline, Yes/No (%) Number of drugs	13.5/86.5 0(0)	30/70 0(0–1)	.134
Drugs at last FUP, yes/no (%) number of drugs	13.5/86.5 0(0)	45/55 1.5(0–2)	** < .001**
Strabismus, yes/no (%)	13.5/86.5	43.3/56.7	**.011**
SER, D	3.28 ± 3.04	6.07 ± 3.89	**.003**

*BCVA* best-corrected VA, *IOP,* intraocular pressure, *AL* axial length, *CDR* cup-to-disk ratio, *SER* spherical equivalent of refraction

## Discussion

In this study, we evaluated the VA of children affected by PCG and compensated by therapy at each year of age and drew a curve of development of VA of this population. Secondly, we assessed the factors associated with a worse visual outcome, dividing the population into two groups based on visual acuity (in or out of interval for age). The study subjects did not follow classic PCG demographics showing a male predominance. Overall, this study addresses a rare disease that is difficult to study at the population level. The incidence of PCG varies according to the geographic area and ethnicity: in western countries, it lies within 1 per 10–20,000 live births [18], while it is higher in the Middle East (1 per 2500 live births), where consanguineous marriages are more prevalent [19, 20]. To date, there are very few studies with a large enough sample size that have examined long-term visual outcomes in children with PCG in European countries [5, 21–23]. Generally, it is challenging to follow the progression of visual function in infancy, especially because children often are not prone to cooperate with the measurement of VA. In the current study, 53.4% of eyes achieved a good VA (< 0.5 LogMAR) and 31.5% had moderate VA (> 0.5 LogMAR and < 1 LogMAR). Our findings are comparable to those of previous studies (34.2–79%) with a similar number of eyes (range 68–108) and of follow-up length (range 1.7–7 years) [5, 21, 24–26]. The rate of complications after IOP-lowering surgical procedures (2.74%) is in line with that of other congenital glaucoma series [5]. A progressive decline in success rate over time was described in various studies [22, 24, 25, 27]. In the attempt to standardize the results of the study and to ease the use of data for the counselling ophthalmologist, we considered age-corrected BCVA and not the BCVA in different time points of follow-up. It is more useful to understand whether patients are below or over the normal range for their age than only knowing BCVA variation in time. Visual acuity may be related to the type of visual acuity testing and could be overestimated at a younger age because, at this age, a preferential looking test was used. Best VA outcomes were reached for the infantile group (Fig. 1), suggesting that there is an influence of the age at onset, and first surgery, in the outcome. This was probably due to the development of the subcortical neural stream or a low degree of embryogenesis in the neonatal group. Longer ALs correlate with a worse VA prognosis, as previously described by Sampaolesi et al. [28]. Amblyopia, irrespective of its subtypes, remains the major cause for VA impairment in controlled postoperative PCG cases, as shown in this study and supported by previous studies [29, 30]. Patients who required multiple surgeries had worse visual results. Unilaterality of the disease did not represent a cause of visual impairment, in contrast with previous studies [27], probably because children with unilateral disease belonged to the infantile group (with a mean age of 24 months), required less surgical interventions and had a better VA prognosis. PCG is a challenging pathology to manage. It requires strict monitoring with short-term follow-up, especially in the first three years of life. An early diagnosis, timely surgical intervention and subsequent rehabilitation are necessary for optimal management of the disease. Up to half of the patients have an unpredictable prognosis and require lifetime monitoring to early recognize the causes of visual loss [31, 32]. Moreover, visual impairment may have a major impact on the child’s development, education, social integration and independence [33, 34]. In conclusion, this study underlines the role of the growth curves in the analysis of the patient’s visual function and the early detection of the causes of low vision for proper management. It also highlights the long-term visual outcomes of PCG at a university teaching hospital in Europe.

## Data Availability

Data are available, if requested.
